# Adaptive Immunity Is the Key to the Understanding of Autoimmune and Paraneoplastic Inflammatory Central Nervous System Disorders

**DOI:** 10.3389/fimmu.2017.00336

**Published:** 2017-03-23

**Authors:** Robert Weissert

**Affiliations:** ^1^Department of Neurology, Neuroimmunology, University of Regensburg, Regensburg, Germany

**Keywords:** T cell, B cell, major histocompatibility complex, human leukocyte antigen, multiple sclerosis, neuromyelitis optica spectrum disorders, autoimmune encephalitis, paraneoplastic disease

## Abstract

There are common aspects and mechanisms between different types of autoimmune diseases such as multiple sclerosis (MS), neuromyelitis optica spectrum disorders (NMOSDs), and autoimmune encephalitis (AE) as well as paraneoplastic inflammatory disorders of the central nervous system. To our present knowledge, depending on the disease, T and B cells as well as antibodies contribute to various aspects of the pathogenesis. Possibly the events leading to the breaking of tolerance between the different diseases are of great similarity and so far, only partially understood. Beside endogenous factors (genetics, genomics, epigenetics, malignancy) also exogenous factors (vitamin D, sun light exposure, smoking, gut microbiome, viral infections) contribute to susceptibility in such diseases. What differs between these disorders are the target molecules of the immune attack. For T cells, these target molecules are presented on major histocompatibility complex (MHC) molecules as MHC-bound ligands. B cells have an important role by amplifying the immune response of T cells by capturing antigen with their surface immunoglobulin and presenting it to T cells. Antibodies secreted by plasma cells that have differentiated from B cells are highly structure specific and can have important effector functions leading to functional impairment or/and lesion evolvement. In MS, the target molecules are mainly myelin- and neuron/axon-derived proteins; in NMOSD, mainly aquaporin-4 expressed on astrocytes; and in AE, various proteins that are expressed by neurons and axons.

## Introduction

Various autoimmune and paraneoplastic disorders of the central nervous system (CNS) share many immunological similarities. In these disorders, immunologic tolerance to self-antigens is broken (1). This failure can be on the T cell as well as on the B cell side or on both sides. The reasons why tolerance is broken in autoimmune diseases are multiple and can differ from paraneoplastic diseases (2). In autoimmune disease, the initial trigger that leads to breaking of tolerance is not as well understood (3). Possibly viral, bacterial and fungal antigens that share antigenic properties with self-antigens can result in activation of T or/and B cells that also recognize self-antigens in the CNS (4). Another possibility could be that in certain autoimmune-prone individuals compared with non-autoimmune-prone individuals, the T and B cell repertoires contain higher quantities of cells with a high avidity for self-antigens that can be activated and can gain access to the CNS in which they find relevant target structures. Such a scenario has been underscored in rodent models of CNS autoimmune diseases (5). In paraneoplastic diseases of the CNS, epitopes from a neoplasm are exposed on antigen-presenting cells to T cells which subsequently also recognize an epitope of similar structural appearance in the CNS (6).

Importantly, the T and B cell epitopes can differ depending on antigen processing in autoimmune disease and possibly also paraneoplastic disease (7, 8). In many autoimmune and paraneoplastic diseases of the CNS, the B cell response is much better characterized as compared with the T cell response (1, 9). T cell help is required for differentiation of B cells into plasma cells and affinity maturation of antibodies (10, 11). There are also B cells which do not require T cell help in autoimmunity, but these do not seem to be of major importance in CNS autoimmunity and CNS paraneoplastic diseases as far as one knows to date (12–14).

## Endogenous Factors

Endogenous factors that contribute to the induction of autoimmunity or paraneoplastic diseases are multiple. First, genetics is of paramount importance. Most autoimmune diseases are complex genetic diseases (15). This means that certain allelic variants of genes predispose to autoimmunity. There are also few examples of autoimmune diseases in which single mutated genes predispose to autoimmunity (16). For CNS-directed autoimmune diseases, no confirmed single genes with mutations have been discovered so far. Much work has been done in elucidating genes that contribute to the complex genetic etiologies (17). Most probably also in CNS immune-directed disorders with paraneoplastic origins, complex genetics are of importance. RNA expression levels have been shown to be altered in autoimmune and paraneoplastic diseases of the CNS (18–20). Tissue with altered RNA expression levels as compared with healthy tissue might predispose to autoimmunity and paraneoplastic diseases (21). There is increased understanding that epigenetics is very crucial in susceptibility to autoimmunity and paraneoplastic disorders (22, 23). Much will be learned in the next years regarding epigenetic regulation of immunity and autoimmunity.

## Exogenous Factors

Much has been discovered regarding exogenous factors that affect autoimmune diseases of the CNS. These exogenous factors cooperate with endogenous factors in susceptibility to autoimmune diseases of the CNS (24). Low vitamin D levels as well as low sun light exposure have been shown to contribute to susceptibility to multiple sclerosis (MS) also independently of other factors (25–27). So far, the influence of vitamin D and sun light exposure has not been defined to the same degree for neuromyelitis optica spectrum disorders (NMOSDs) and autoimmune encephalitis (AE) (28). Smoking has a negative influence on MS (29, 30). This influence is controlled to some degree by human leukocyte antigen (HLA) genes underscoring that HLA-presented autoantigens are possibly modified and promote a more vigorous autoimmune response (29). It has been shown that in MS there is a change of the gut microbiome (31, 32). Also in NMOSD, changes in the gut microbiome have been observed with overrepresentation of *Clostridium perfringens* (33). In experimental autoimmune encephalomyelitis (EAE), it has been experimentally proven that the gut microbiome contributes to disease susceptibility (34). So far, in most types of diseases it is not well defined what specific bacteria of the gut microbiome drive autoimmune disease. It has been shown that Epstein–Barr virus (EBV) infection has an influence on MS susceptibility (35, 36). The influence is mainly mediated in childhood and most likely affects the T cell repertoire. Even though a direct role of EBV infection in MS lesion development was claimed, this could not be confirmed (37). Also, salt intake has been shown to influence EAE susceptibility (38). So far, it is not clear if levels of salt intake are influencing susceptibility or disease course in MS (39). The elucidation of the influence of nutritional factors in various autoimmune diseases of the CNS is presently investigated in more detail. Regarding paraneoplastic diseases, no such influence has been elucidated so far.

## Major Histocompatibility Complex (MHC)/HLA Haplotypes

Most autoimmune diseases are associated with certain MHC/HLA haplotypes (40). Such associations also exist for some paraneoplastic diseases such as paraneoplastic pemphigus (41). So far, influences of HLA haplotypes on paraneoplastic diseases have not been investigated in much detail. The reason for the haplotype preferences of specific autoimmune diseases is not known.

The most likely scenario for influences of HLA haplotypes on autoimmune diseases indicates that during early tolerance development certain HLA haplotypes select for a T cell repertoire that can be self-biased to certain autoantigens and certain organs (42–44). In the emergence of tolerance, there is selection of a broad range of T cell receptors (TCRs) on various self-antigens. In a first step, only T cells are selected that recognize self MHC-peptide complexes (45–47). In the next step, T cells with TCRs with a too high affinity for such complexes are deleted from the repertoire (48, 49). MHC displayed peptide repertoires influence positive and negative selection (50). Based on the expressed HLA haplotypes, the predetermined T cell repertoire differs in individuals (51, 52). The TCR repertoire has a bias depending on the HLA haplotype in avidity for certain self-antigens (53, 54).

In MHC congenic rat strains, we have shown that there is an autoantigen preference that can result, depending on the expressed MHC alleles, in disease susceptibility or protection from certain diseases (5, 55). Interestingly with increasing complexity of the disease driving autoantigen, the MHC haplotype-dependent effects alleviate (56, 57). Also, we have shown that the amount of autoantigen that leads to disease induction can differ between different MHC haplotypes (5, 58). This means that in one MHC haplotype minute amounts of antigens are sufficient to induce severe disease, while in others much higher amounts would be necessary. These findings underscore the influence of the antigenic load in context with genetic factors. It has been shown that depending on the expressed MHC haplotype, the cytokine preference of the selected T cell repertoire differs (44, 59). Recently in an experimental model of rheumatoid arthritis (RA), it has been shown that MHC alleles that drive disease are associated with a T helper cell type 1 (Th1) response with secretion of interferon-gamma (IFN-γ) (60). By contrast, protective MHC alleles promoted an interleukin-17 T helper (Th17) cell response. Such a predetermination of cytokine responses to disease-inducing factors is potentially also shaped early in tolerance development and can also contribute to the finding that certain HLA haplotypes predispose to certain autoimmune diseases while others protect from disease.

## Neoantigens

Tolerance can be broken by presentation of neoantigens on MHC molecules to T cells recognizing antigens that share structural similarities to self-molecules (61). Recently, it has been shown that neoantigens for presentation on MHC I molecules can be generated by fusion of different fragments of degraded proteins during antigen processing (62). In addition, endogenous neoantigens could evolve by mutation or translational defects. So far, the experimental data that such novel antigens could play a role in the induction or maintenance of autoimmune disease of the CNS are still lacking but an interesting avenue of future research efforts.

Posttranslational modifications of antigens can also lead to induction of autoimmunity (63). This has been shown for RA in which citrullinated epitopes have been shown to be disease inducing (64, 65). Also for MS, a role for citrullination has been proposed but so far there is no proof for the relevance in the experimental or human setting (66, 67). Possibly transpeptidation could be of importance as has been shown in a model of diabetes (68). Changes in glycosylation can affect induction of autoimmunity (69). Also, other types of posttranslational modifications could be of great relevance but have not been investigated in much detail regarding CNS autoimmunity or CNS paraneoplastic diseases. We have shown that even the conformational state of an autoantigen can have different consequences on disease induction capacity (8). Therefore, different conformations of an antigen can be seen be the immune system in a “neoantigenic” fashion and lead to autoimmunity (70).

## Specific Diseases

### Multiple Sclerosis

In MS, the target of the autoimmune response, which seems to be predominantly T cell driven, is mainly directed against proteins of the myelin sheath which is produced by oligodendrocytes (1) (Table 1). Myelin basic protein (MBP) is thought to be the major autoantigen which is involved (71, 72). Many researchers have addressed this topic and found additional myelin proteins that can be the target of the autoimmune response (1). There are strong indications that the humoral immune response is important as well (73). Nevertheless, the exact autoantigens driving this B cell response are not known to date in detail. Myelin oligodendrocyte glycoprotein (MOG) is a model antigen which has been shown to be of major importance driving the B cells response in rodent and primate models (74). This protein, which is expressed on the outer surface of the myelin sheath, seems to be involved in children but not to the same extent in older people with MS in the immune pathogenesis of MS (75). Especially young children with MS with an age under 10 years have a robust anti-MOG antibody response. This finding underscores that potentially early in life immunological events are taking place that predispose to development of MS later in life. CNS lesions of MS patients show antibody-dependent complement destruction underscoring the importance of the antibody response in MS (73). Moreover, proteins expressed on neurons and axons have also been discussed to be targets of the immune response in patients with MS based on work in EAE (76). Recently, in patients with MS, we have shown that peptides can be eluted from MHC molecules from CNS tissue that are recognized by T cells secreting IFN-γ (Th1) (72, 77). Importantly, the increased immune reactivity against such peptides is observed in patients with active MS, i.e., in patients with MS who have an acute bout- or/and contrast-enhancing lesions in the CNS indicating active inflammation. This finding underscores that the adaptive immune response against CNS-derived autoantigens is of significance in MS. Importantly, the T cell reactivity is directed not only against MBP but additional autoantigens and differs between individuals.

**Table 1:** **Human diseases and autoantigens, main cellular expression, and cellular compartment of expression as well as involved immune responses as presently known**.

Disease	(Auto)antigen	Target cell	Main cellular localization	Established role of		
				T cells	Antibodies	Complement
MS	Actin	U	C, CS, ES	+ (72)	ND	ND
MS	Alpha-synuclein	N	U, not P	+ (72)	ND	ND
MS	CNPase, 2′,3′-cyclic-nucleotide 3′-phosphodiesterase	O, N	ES, CS, N	+ (78)	+ (79)	ND
MS	GFAP, glial fibrillary acidic protein	A	C, CS	+ (72)	− (80)	ND
MS	Glutamate dehydrogenase	U	M	+ (72)	ND	ND
MS	MAG, myelin-associated glycoprotein	O	PM	+ (81)	+ (81)	ND
MS	MBP, myelin basic protein	O	PM, C, N	+ (72, 82, 83)	+ (79)	ND
MS	MOBP, myelin-associated oligodendrocyte basic protein	O	PM	+ (84)	+ (79)	ND
MS	MOG, myelin oligodendrocyte glycoprotein	O	PM	+ (85, 86)	+ (87)	+ (88)
MS	Neurofilament-3	N	CS, C, N	+ (72)	+ (89)	ND
MS	PLP, proteolipid protein	O	PM	+ (90)	+ (79)	ND
MS	S100β, S100 calcium-binding protein B	A	E, C, N	+ (91)	− (80)	ND
MS	Survivin	U	C, CS, N	+ (72)	ND	ND
MS	Transaldolase	U	E, C, N	+ (92)	+ (93)	ND
NMOSD	AQP4, aquaporin-4	A	PM	+ (94)	+ (95)	+ (96)
NMOSD	MOG, myelin oligodendrocyte glycoprotein	O	PM	ND	+ (97)	+ (88)
AE	AK5, adenylate kinase 5	N	C, ES	ND	+ (98)	ND
AE	AMPAR, glutamate ionotropic receptor AMPA type	N	PM	ND	+ (99)	ND
AE	Amphiphysin	N	PM, C, CS, GA	ND	+ (100)	ND
AE	CASPR2, contactin associated protein-like 2	N	PM, E, GA	ND	+ (101)	ND
AE	CRMP5, dihydropyrimidinase-like 5	N	C	ND	+ (102)	ND
AE	DNER (Tr), delta-/notch-like EGF repeat containing	N	PM, E	ND	+ (103)	ND
AE	Dopamine receptor D2	N	PM, C	ND	+ (104)	ND
AE	DPPX, dipeptidyl peptidase	N	ES, L, PM, V	ND	+ (105)	ND
AE	GABAaR, gamma-aminobutyric acid type A receptor	N	PM	ND	+ (106)	ND
AE	GABAbR, gamma-aminobutyric acid type B receptor	N	PM	ND	+ (107)	ND
AE	GAD65, glutamate decarboxylase 2	N	C, PM	+ (108)	+ (109)	− (108)
AE	GlyR, glycine receptor	N	PM	ND	+ (110)	ND
AE	Hu, ELAV-like RNA-binding protein 4	N	C, N	+ (108, 111)	+ (112)	− (108)
AE	IgLON5, IgLON family member 5	N	ES, PM	ND	+ (113)	ND
AE	LGI1, leucine-rich glioma-inactivated 1	N	ES, PM	+ (108)	+ (114, 115)	+ (108)
AE	Ma1, paraneoplastic Ma antigen 1	N	N	ND	+ (116)	ND
AE	Ma2, paraneoplastic Ma antigen 2	N	N	+ (108)	+ (117)	− (108)
AE	mGluR1, glutamate metabotropic receptor 1	N	PM, C	ND	+ (118)	ND
AE	mGluR5, glutamate metabotropic receptor 5	N	ES, PM	ND	+ (119)	ND
AE	Neurexin-3a	N	PM	ND	+ (120)	ND
AE	NMDAR, glutamate ionotropic receptor NMDA type	N	PM	+ (108)	+ (121)	− (108)
AE	P/Q type VGCC, calcium voltage-gated channel	N	PM	ND	+ (122)	ND
AE	Ri, NOVA alternative splicing regulator 1	N	N	ND	+ (123)	ND
AE	Yo, cerebellar degeneration-related protein 2	N	N	ND	+ (124, 125)	ND
AE	Zic4, Zic family member 4	N	N	ND	+ (126)	ND

*A, astrocytes; AE, autoimmune encephalitis; C, cytosol; CS, cytoskeleton; E, endosome; ES, extracellular space; GA, Golgi apparatus; L, lysosome; M, mitochondria; MS, multiple sclerosis; N, neurons; N, nucleus; ND, not determined; NMOSD, neuromyelitis optica spectrum disorder; O, oligodendrocytes; P, peroxisome; PM, plasma membrane; U, ubiquitous; V, vacuoles; +, positive findings; −, negative findings*.

### Neuromyelitis Optica Spectrum Disorders

In NMOSD, it has been demonstrated that the immune response is targeting aquaporin-4 (AQP4), a water channel protein on astrocytes (127) (Table 1). Certain cases of NMOSD are associated with an immune response against MOG (128). In both types, antibody-dependent tissue destruction is of major importance (96). The role of T cells is presently analyzed in more detail (94). In rodent models, it has been delineated that direct injection of anti-AQP4 antibody in the CNS can lead to severe pathology without the presence of T cells (129). Also, antibody-dependent destruction of tissue by complement seems to be of paramount importance and dependent on the antigen conformation and the presence of antibodies (130). It is not excluded that also additional target molecules will be discovered, which are associated with seronegative forms of NMOSD in the future.

### Autoimmune Encephalitis

There are a high number of diseases in which the autoimmune response is directed against neuronal antigens (1) (Table 1). The target molecules can be localized intracellular or extracellular (9, 98). Some of the intracellular antigens are nuclear proteins. Most of the diseases in which the immune response is directed against intracellular neuronal targets are of paraneoplastic origin. Mainly, CD8+ T cells, which are MHC I restricted, are involved in the immune pathogenesis of these types of AE (131). In affected patients, most important is the search for the underlying neoplasm and its treatment. In addition, immunotherapy is meaningful (132).

In diseases in which the target structures are exposed extracellular as membrane proteins, more diseases are of autoimmune origin and less paraneoplastic. A prototype is the anti-*N*-methyl-d-aspartate receptor (NMDA) receptor encephalitis in which the NMDA receptor is the target molecule of the immune response (121). It has been shown that antibodies are most important in this type of diseases and that these antibodies can lead to alteration of cellular function with consequences on behavior (104, 133, 134) or tissue destruction by complement (108). There is a requirement for these antibodies to access the CNS in order to be of disease relevance (135). Recently, a strong influence of a specific HLA haplotype has been shown in anti-LGI1 encephalitis in Koreans (136). So far, the role of T cells has not been assessed in detail in these illnesses but deserves much more attention in future efforts. These diseases are treated by immunotherapy (132, 137). In cases in which the origin is paraneoplastic, the neoplasm needs to be treated in addition to immunotherapy.

### Paraneoplastic Disease of the CNS

Why is there such a preference of paraneoplastic CNS disorders for neuronal antigens? Why are there no or only few cases of paraneoplastic MS or NMOSD? Possibly the answer lies in the antigen repertoires that are preferentially displayed by neoplasms. In NMOSD, cases with paraneoplastic origin have been reported (138, 139). In addition, certain brain neoplasms might result in paraneoplastic cases of MS even though such cases have not been interpreted as paraneoplastic diseases so far but rather in the opposite way that the molecular changes in the MS lesion have led to development of the neoplasms (140–142). As discussed in the section regarding HLA haplotypes, the density of the presented disease-inducing antigen expressed by the neoplasms is potentially an important factor that can lead to paraneoplastic disease. Therefore, a higher density of the presented autoantigen would possibly more likely lead to disease induction.

## Therapeutic Considerations

Since the adaptive immune response is of such great relevance in various immunologically mediated disorder of the CNS it is obvious that it should be targeted to halt and possibly cure autoimmune and paraneoplastic diseases of the CNS. Of course, in paraneoplastic diseases always the underlying malignancy should be treated by surgical, radiotherapeutic, and chemotherapeutic approaches, since the eradication of the malignancy with presence of the antigens that drive the disease can possibly lead to an improvement of the paraneoplastic disease condition affecting the CNS. It has been proposed that immunotherapeutic approaches should mainly affect the humoral immune response, since the cellular immune response by CD8+ T cells is of great importance in tumor rejection (143). This aspect needs to be investigated in more depth.

In autoimmune diseases of CNS depending on the dominance of the T or/and B cell response, a rational treatment approach should be used. In most diseases in which autoantibodies are of major importance, the depletion of B cells by rituximab a monoclonal antibody (mAb) that targets CD20 has been shown to be of great efficacy (144–146). This is the case for AE with membrane molecules as target antigens of the immune response (132, 137, 146). In NMOSD, depletion of B cells is well established as a very efficacious treatment approach (145). Also in MS, depletion of B cells has been shown to be of great therapeutic efficacy (144). This has been underscored by recent data with ocrelizumab a novel human mAb also targeting CD20 (147, 148). Since B cells are very potent professional antigen-presenting cells, the depletion of such cells leads also to reduced presentation of antigens to T cells (1, 149, 150). This reduction of antigen presentation in individuals that have been treated with B cell depleting agents is potentially one of the most important immunotherapeutic effects of such a therapeutic approach.

In MS, it has been demonstrated that decreasing numbers of T cells that enter the CNS can result in reduction of contrast enhancing lesions, numbers of new lesions, and improvement of clinical disease score as well (3). Also, modulating T cell responses regarding the way how these cells react in expression of certain immune mediators can affect disease.

The combined depletion of T and B cells by alemtuzumab by targeting CD52 has been shown to be very efficacious in MS (151–153). In a retrospective case series in NMOSD, this approach failed to be effective (154). The reasons are not clear so far, but the authors recommend caution. The approach has not been used in AE so far. This restricted use is most likely because potential side effects are dreaded. Nevertheless, such a therapeutic approach embodies a great potential for cure in selected patient populations.

Another approach to affect autoimmune and paraneoplastic diseases would be the blockade of the terminal phase of inflammation which is partially mediated by antibodies and that leads to tissue destruction. In this aspect, the use of eculizumab, a mAb depleting the complement factor C5 holds great promise. There are trials ongoing in NMOSD that investigate the efficacy of eculizumab in disease arrest. Initial observations are very promising (155). The use of complement inhibitors could be of great therapeutic potential in MS as well as in certain types of AE in which complement is strongly involved in pathophysiology. So far, the use of eculizumab is restricted due to limited clinical development efforts because of its high cost.

## Conclusion

**Figure 1:**
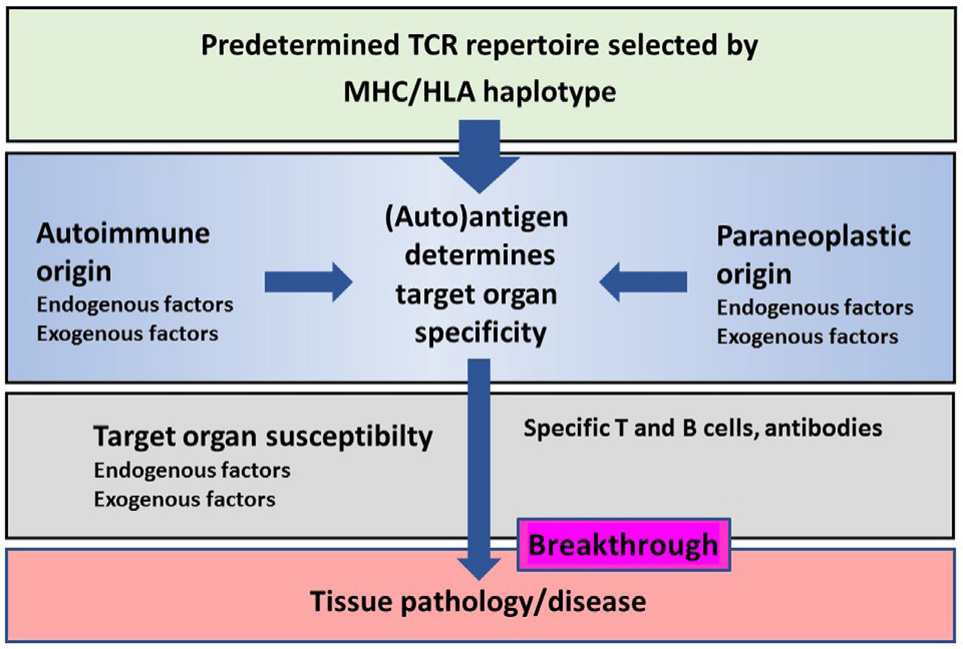
**Schematic representation of the major histocompatibility complex (MHC)/human leukocyte antigen (HLA) haplotype and additional factors on disease breakthrough in autoimmune or/and paraneoplastic diseases**.

There is a great similarity in immune mechanisms of different autoimmune and paraneoplastic diseases of the CNS (Figure 1). The adaptive immunity seems to be the main driver of selected organ pathology in autoimmune and paraneoplastic diseases. Specific HLA haplotypes are associated with different autoimmune and paraneoplastic autoimmune disorders. HLA haplotypes predispose for selection of certain autoantigens that drive such diseases. The phenotype of autoimmune and paraneoplastic diseases of the CNS differs depending on the antigens that drive the immune responses. Neoantigens can possibly contribute to the development of these disorders. The pivotal role of the adaptive immunity in autoimmune and paraneoplastic diseases of the CNS allows directed immune interventions to modulate T and B cell responses.

## Author Contributions

RW outlined the subject of the review; searched for, analyzed, and interpreted the literature; wrote the manuscript, and agreed to be accountable for all aspects of the work.

## Conflict of Interest Statement

The author declares that the research was conducted in the absence of any commercial or financial relationships that could be construed as a potential conflict of interest.

## References

[1] Riedhammer C Weissert R. Antigen presentation, autoantigens, and immune regulation in multiple sclerosis and other autoimmune diseases. Front Immunol (2015) 6:322. 10.3389/fimmu.2015.00322 26136751 PMC4470263

[2] Maverakis E Goodarzi H Wehrli LN Ono Y Garcia MS The etiology of paraneoplastic autoimmunity. Clin Rev Allerg Immunol (2012) 42(2):135–44. 10.1007/s12016-010-8248-5 21246308

[3] Weissert R. The immune pathogenesis of multiple sclerosis. J Neuroimmune Pharmacol (2013) 8(4):857–66. 10.1007/s11481-013-9467-3 23660832

[4] Wucherpfennig KW Strominger JL. Molecular mimicry in T cell-mediated autoimmunity: viral peptides activate human T cell clones specific for myelin basic protein. Cell (1995) 80(5):695–705. 10.1016/0092-8674(95)90348-8 7534214 PMC7133435

[5] Weissert R Wallstrom E Storch MK Stefferl A Lorentzen J Lassmann H MHC haplotype-dependent regulation of MOG-induced EAE in rats. J Clin Invest (1998) 102(6):1265–73. 10.1172/JCI3022 9739061 PMC509110

[6] Posner JB. Pathogenesis of central nervous system paraneoplastic syndromes. Rev Neurol (Paris) (1992) 148(6–7):502–12. 1280371

[7] Weissert R de Graaf KL Storch MK Barth S Linington C Lassmann H MHC class II-regulated central nervous system autoaggression and T cell responses in peripheral lymphoid tissues are dissociated in myelin oligodendrocyte glycoprotein-induced experimental autoimmune encephalomyelitis. J Immunol (2001) 166(12):7588–99. 10.4049/jimmunol.166.12.7588 11390515

[8] de Graaf KL Albert M Weissert R. Autoantigen conformation influences both B- and T-cell responses and encephalitogenicity. J Biol Chem (2012) 287(21):17206–13. 10.1074/jbc.M111.304246 22493449 PMC3366859

[9] Dalmau J. NMDA receptor encephalitis and other antibody-mediated disorders of the synapse: the 2016 Cotzias Lecture. Neurology (2016) 87(23):2471–82. 10.1212/WNL.0000000000003414 27920282 PMC5177671

[10] Allen CD Okada T Tang HL Cyster JG. Imaging of germinal center selection events during affinity maturation. Science (2007) 315(5811):528–31. 10.1126/science.1136736 17185562

[11] Weinstein JS Hernandez SG Craft J. T cells that promote B-Cell maturation in systemic autoimmunity. Immunol Rev (2012) 247(1):160–71. 10.1111/j.1600-065X.2012.01122.x 22500839 PMC3334351

[12] Shlomchik MJ. Activating systemic autoimmunity: B’s, T’s, and tolls. Curr Opin Immunol (2009) 21(6):626–33. 10.1016/j.coi.2009.08.005 19800208 PMC2787881

[13] Claes N Fraussen J Stinissen P Hupperts R Somers V. B cells are multifunctional players in multiple sclerosis pathogenesis: insights from therapeutic interventions. Front Immunol (2015) 6:642. 10.3389/fimmu.2015.00642 26734009 PMC4685142

[14] von Budingen HC Bar-Or A Zamvil SS. B cells in multiple sclerosis: connecting the dots. Curr Opin Immunol (2011) 23(6):713–20. 10.1016/j.coi.2011.09.003 21983151 PMC4188435

[15] Richard-Miceli C Criswell LA. Emerging patterns of genetic overlap across autoimmune disorders. Genome Med (2012) 4(1):6. 10.1186/gm305 22284131 PMC3334554

[16] Tadaki H Saitsu H Nishimura-Tadaki A Imagawa T Kikuchi M Hara R De novo 19q13.42 duplications involving NLRP gene cluster in a patient with systemic-onset juvenile idiopathic arthritis. J Hum Genet (2011) 56(5):343–7. 10.1038/jhg.2011.16 21326309

[17] International Multiple Sclerosis Genetics Consortium, Wellcome Trust Case Control Consortium Sawcer S Hellenthal G Pirinen M Spencer CC Genetic risk and a primary role for cell-mediated immune mechanisms in multiple sclerosis. Nature (2011) 476(7359):214–9. 10.1038/nature10251 21833088 PMC3182531

[18] Lock C Hermans G Pedotti R Brendolan A Schadt E Garren H Gene-microarray analysis of multiple sclerosis lesions yields new targets validated in autoimmune encephalomyelitis. Nat Med (2002) 8(5):500–8. 10.1038/nm0502-500 11984595

[19] Herrmann MM Barth S Greve B Schumann KM Bartels A Weissert R. Identification of gene expression patterns crucially involved in experimental autoimmune encephalomyelitis and multiple sclerosis. Dis Model Mech (2016) 9(10):1211–20. 10.1242/dmm.025536 27519689 PMC5087830

[20] Ince-Dunn G Okano HJ Jensen KB Park WY Zhong R Ule J Neuronal Elav-like (Hu) proteins regulate RNA splicing and abundance to control glutamate levels and neuronal excitability. Neuron (2012) 75(6):1067–80. 10.1016/j.neuron.2012.07.009 22998874 PMC3517991

[21] Hathaway CK Chang AS Grant R Kim HS Madden VJ Bagnell CR Jr High Elmo1 expression aggravates and low Elmo1 expression prevents diabetic nephropathy. Proc Natl Acad Sci U S A (2016) 113(8):2218–22. 10.1073/pnas.1600511113 26858454 PMC4776516

[22] Huynh JL Garg P Thin TH Yoo S Dutta R Trapp BD Epigenome-wide differences in pathology-free regions of multiple sclerosis-affected brains. Nat Neurosci (2014) 17(1):121–30. 10.1038/nn.3588 24270187 PMC3934491

[23] Boumahdi S Driessens G Lapouge G Rorive S Nassar D Le Mercier M SOX2 controls tumour initiation and cancer stem-cell functions in squamous-cell carcinoma. Nature (2014) 511(7508):246–50. 10.1038/nature13305 24909994

[24] Olsson T Barcellos LF Alfredsson L. Interactions between genetic, lifestyle and environmental risk factors for multiple sclerosis. Nat Rev Neurol (2017) 13(1):25–36. 10.1038/nrneurol.2016.187 27934854

[25] Becklund BR Severson KS Vang SV DeLuca HF. UV radiation suppresses experimental autoimmune encephalomyelitis independent of vitamin D production. Proc Natl Acad Sci U S A (2010) 107(14):6418–23. 10.1073/pnas.1001119107 20308557 PMC2851981

[26] Rhead B Baarnhielm M Gianfrancesco M Mok A Shao X Quach H Mendelian randomization shows a causal effect of low vitamin D on multiple sclerosis risk. Neurol Genet (2016) 2(5):e97. 10.1212/NXG.0000000000000097 27652346 PMC5022843

[27] Islam T Gauderman WJ Cozen W Mack TM. Childhood sun exposure influences risk of multiple sclerosis in monozygotic twins. Neurology (2007) 69(4):381–8. 10.1212/01.wnl.0000268266.50850.48 17646631

[28] Min JH Waters P Vincent A Cho HJ Joo BE Woo SY Low levels of vitamin D in neuromyelitis optica spectrum disorder: association with disease disability. PLoS One (2014) 9(9):e107274. 10.1371/journal.pone.0107274 25211011 PMC4161425

[29] Hedstrom AK Sundqvist E Baarnhielm M Nordin N Hillert J Kockum I Smoking and two human leukocyte antigen genes interact to increase the risk for multiple sclerosis. Brain (2011) 134(Pt 3):653–64. 10.1093/brain/awq371 21303861

[30] Hedstrom AK Baarnhielm M Olsson T Alfredsson L. Tobacco smoking, but not Swedish snuff use, increases the risk of multiple sclerosis. Neurology (2009) 73(9):696–701. 10.1212/WNL.0b013e3181b59c40 19720976

[31] Chen J Chia N Kalari KR Yao JZ Novotna M Soldan MM Multiple sclerosis patients have a distinct gut microbiota compared to healthy controls. Sci Rep (2016) 6:28484. 10.1038/srep28484 27346372 PMC4921909

[32] Jangi S Gandhi R Cox LM Li N von Glehn F Yan R Alterations of the human gut microbiome in multiple sclerosis. Nat Commun (2016) 7:12015. 10.1038/ncomms12015 27352007 PMC4931233

[33] Cree BA Spencer CM Varrin-Doyer M Baranzini SE Zamvil SS. Gut microbiome analysis in neuromyelitis optica reveals overabundance of *Clostridium perfringens*. Ann Neurol (2016) 80(3):443–7. 10.1002/ana.24718 27398819 PMC5053302

[34] Berer K Mues M Koutrolos M Rasbi ZA Boziki M Johner C Commensal microbiota and myelin autoantigen cooperate to trigger autoimmune demyelination. Nature (2011) 479(7374):538–41. 10.1038/nature10554 22031325

[35] Ascherio A Munger KL Lennette ET Spiegelman D Hernan MA Olek MJ Epstein–Barr virus antibodies and risk of multiple sclerosis: a prospective study. JAMA (2001) 286(24):3083–8. 10.1001/jama.286.24.3083 11754673

[36] Levin LI Munger KL O’Reilly EJ Falk KI Ascherio A. Primary infection with the Epstein–Barr virus and risk of multiple sclerosis. Ann Neurol (2010) 67(6):824–30. 10.1002/ana.21978 20517945 PMC3089959

[37] Willis SN Stadelmann C Rodig SJ Caron T Gattenloehner S Mallozzi SS Epstein–Barr virus infection is not a characteristic feature of multiple sclerosis brain. Brain (2009) 132(Pt 12):3318–28. 10.1093/brain/awp200 19638446 PMC2792367

[38] Kleinewietfeld M Manzel A Titze J Kvakan H Yosef N Linker RA Sodium chloride drives autoimmune disease by the induction of pathogenic TH17 cells. Nature (2013) 496(7446):518–22. 10.1038/nature11868 23467095 PMC3746493

[39] Nourbakhsh B Graves J Casper TC Lulu S Waldman A Belman A Dietary salt intake and time to relapse in paediatric multiple sclerosis. J Neurol Neurosurg Psychiatry (2016) 87(12):1350–3. 10.1136/jnnp-2016-313410 27343226 PMC5370574

[40] Klein J Sato A The HLA system. First of two parts. N Engl J Med (2000) 343(10):702–9. 10.1056/NEJM200009073431006 10974135

[41] Martel P Loiseau P Joly P Busson M Lepage V Mouquet H Paraneoplastic pemphigus is associated with the DRB1*03 allele. J Autoimmun (2003) 20(1):91–5. 10.1016/S0896-8411(02)00092-6 12604316

[42] Segurado OG Arnaiz-Villena A Wank R Schendel DJ. The multifactorial nature of MHC-linked susceptibility to insulin-dependent diabetes. Autoimmunity (1993) 15(1):85–9. 10.3109/08916939309004844 8218835

[43] Abraham RS Kudva YC Wilson SB Strominger JL David CS. Co-expression of HLA DR3 and DQ8 results in the development of spontaneous insulitis and loss of tolerance to GAD65 in transgenic mice. Diabetes (2000) 49(4):548–54. 10.2337/diabetes.49.4.548 10871191

[44] Murray JS Madri J Tite J Carding SR Bottomly K. MHC control of CD4+ T cell subset activation. J Exp Med (1989) 170(6):2135–40. 10.1084/jem.170.6.2135 2573684 PMC2189542

[45] MacDonald HR Lees RK Schneider R Zinkernagel RM Hengartner H. Positive selection of CD4+ thymocytes controlled by MHC class II gene products. Nature (1988) 336(6198):471–3. 10.1038/336471a0 3264054

[46] Kappler J Kotzin B Herron L Gelfand EW Bigler RD Boylston A V beta-specific stimulation of human T cells by staphylococcal toxins. Science (1989) 244(4906):811–3. 10.1126/science.2524876 2524876

[47] Sha WC Nelson CA Newberry RD Kranz DM Russell JH Loh DY. Positive and negative selection of an antigen receptor on T cells in transgenic mice. Nature (1988) 336(6194):73–6. 10.1038/336073a0 3263574

[48] Hengartner H Odermatt B Schneider R Schreyer M Walle G MacDonald HR Deletion of self-reactive T cells before entry into the thymus medulla. Nature (1988) 336(6197):388–90. 10.1038/336388a0 3264053

[49] Gascoigne NR Rybakin V Acuto O Brzostek J TCR signal strength and T cell development. Annu Rev Cell Dev Biol (2016) 32:327–48. 10.1146/annurev-cellbio-111315-125324 27712102

[50] Kincaid EZ Murata S Tanaka K Rock KL. Specialized proteasome subunits have an essential role in the thymic selection of CD8(+) T cells. Nat Immunol (2016) 17(8):938–45. 10.1038/ni.3480 27294792 PMC4955723

[51] Gulwani-Akolkar B Posnett DN Janson CH Grunewald J Wigzell H Akolkar P T cell receptor V-segment frequencies in peripheral blood T cells correlate with human leukocyte antigen type. J Exp Med (1991) 174(5):1139–46. 10.1084/jem.174.5.1139 1940794 PMC2118988

[52] Akolkar PN Gulwani-Akolkar B Pergolizzi R Bigler RD Silver J. Influence of HLA genes on T cell receptor V segment frequencies and expression levels in peripheral blood lymphocytes. J Immunol (1993) 150(7):2761–73. 8454853

[53] Moudgil KD Sercarz EE. Dominant determinants in hen eggwhite lysozyme correspond to the cryptic determinants within its self-homologue, mouse lysozyme: implications in shaping of the T cell repertoire and autoimmunity. J Exp Med (1993) 178(6):2131–8. 10.1084/jem.178.6.2131 8245785 PMC2191305

[54] Morgan DJ Nugent CT Raveney BJ Sherman LA In a transgenic model of spontaneous autoimmune diabetes, expression of a protective class II MHC molecule results in thymic deletion of diabetogenic CD8+ T cells. J Immunol (2004) 172(2):1000–8. 10.4049/jimmunol.172.2.1000 14707073

[55] Weissert R, editor. Experimental autoimmune encephalomyelitis. Models, Disease Biology and Experimental Therapy. Rijeka: In Tech (2012). p. 1–19.

[56] Gaertner S de Graaf KL Olsson T Weissert R. Immunogenicity of Torpedo acetylcholine receptor in the context of different rat MHC class II haplotypes and non-MHC genomes. Immunogenetics (2004) 56(1):61–4. 10.1007/s00251-004-0656-3 15004728

[57] Gaertner S de Graaf KL Wienhold W Wiesmuller KH Melms A Weissert R. Lack of pathogenicity of immunodominant T and B cell determinants of the nicotinic acetylcholine receptor epsilon-chain. J Neuroimmunol (2004) 152(1–2):44–56. 10.1016/j.jneuroim.2004.03.019 15223236

[58] de Graaf KL Berne GP Herrmann MM Hansson GK Olsson T Weissert R. CDR3 sequence preference of TCRBV8S2+ T cells within the CNS does not reflect single amino acid dependent avidity expansion. J Neuroimmunol (2005) 166(1–2):47–54. 10.1016/j.jneuroim.2005.05.004 15963573

[59] Mangalam AK Taneja V David CS. HLA class II molecules influence susceptibility versus protection in inflammatory diseases by determining the cytokine profile. J Immunol (2013) 190(2):513–8. 10.4049/jimmunol.1201891 23293357 PMC3545203

[60] Tuncel J Haag S Holmdahl R. MHC class II alleles associated with Th1 rather than Th17 type immunity drive the onset of early arthritis in a rat model of rheumatoid arthritis. Eur J Immunol (2016). 10.1002/eji.201646760 28012172 PMC7163699

[61] Lambe T Leung JC Bouriez-Jones T Silver K Makinen K Crockford TL CD4 T cell-dependent autoimmunity against a melanocyte neoantigen induces spontaneous vitiligo and depends upon Fas-Fas ligand interactions. J Immunol (2006) 177(5):3055–62. 10.4049/jimmunol.177.5.3055 16920942

[62] Liepe J Marino F Sidney J Jeko A Bunting DE Sette A A large fraction of HLA class I ligands are proteasome-generated spliced peptides. Science (2016) 354(6310):354–8. 10.1126/science.aaf4384 27846572

[63] Anderton SM. Post-translational modifications of self antigens: implications for autoimmunity. Curr Opin Immunol (2004) 16(6):753–8. 10.1016/j.coi.2004.09.001 15511669

[64] Burkhardt H Sehnert B Bockermann R Engstrom A Kalden JR Holmdahl R. Humoral immune response to citrullinated collagen type II determinants in early rheumatoid arthritis. Eur J Immunol (2005) 35(5):1643–52. 10.1002/eji.200526000 15832289

[65] Uysal H Bockermann R Nandakumar KS Sehnert B Bajtner E Engstrom A Structure and pathogenicity of antibodies specific for citrullinated collagen type II in experimental arthritis. J Exp Med (2009) 206(2):449–62. 10.1084/jem.20081862 19204106 PMC2646582

[66] Carrillo-Vico A Leech MD Anderton SM. Contribution of myelin autoantigen citrullination to T cell autoaggression in the central nervous system. J Immunol (2010) 184(6):2839–46. 10.4049/jimmunol.0903639 20164413

[67] Bradford CM Ramos I Cross AK Haddock G McQuaid S Nicholas AP Localisation of citrullinated proteins in normal appearing white matter and lesions in the central nervous system in multiple sclerosis. J Neuroimmunol (2014) 273(1–2):85–95. 10.1016/j.jneuroim.2014.05.007 24907905

[68] Jin N Wang Y Crawford F White J Marrack P Dai S N-terminal additions to the WE14 peptide of chromogranin A create strong autoantigen agonists in type 1 diabetes. Proc Natl Acad Sci U S A (2015) 112(43):13318–23. 10.1073/pnas.1517862112 26453556 PMC4629350

[69] Backlund J Treschow A Bockermann R Holm B Holm L Issazadeh-Navikas S Glycosylation of type II collagen is of major importance for T cell tolerance and pathology in collagen-induced arthritis. Eur J Immunol (2002) 32(12):3776–84. 10.1002/1521-4141(200212)32:12<3776::AID-IMMU3776>3.0.CO;2-A 12516572

[70] Levine SM Raben N Xie D Askin FB Tuder R Mullins M Novel conformation of histidyl-transfer RNA synthetase in the lung: the target tissue in Jo-1 autoantibody-associated myositis. Arthritis Rheum (2007) 56(8):2729–39. 10.1002/art.22790 17665459

[71] Bielekova B Goodwin B Richert N Cortese I Kondo T Afshar G Encephalitogenic potential of the myelin basic protein peptide (amino acids 83-99) in multiple sclerosis: results of a phase II clinical trial with an altered peptide ligand. Nat Med (2000) 6(10):1167–75. 10.1038/80516 11017150

[72] Riedhammer C Halbritter D Weissert R Increased immune reactivity to central nervous system-derived naturally presented peptides in patients with active multiple sclerosis. J Allergy Clin Immunol (2017) 139(2):694–6.e7. 10.1016/j.jaci.2016.08.015 27639936

[73] Storch MK Piddlesden S Haltia M Iivanainen M Morgan P Lassmann H. Multiple sclerosis: in situ evidence for antibody- and complement-mediated demyelination. Ann Neurol (1998) 43(4):465–71. 10.1002/ana.410430409 9546327

[74] Lassmann H Bradl M Multiple sclerosis: experimental models and reality. Acta Neuropathol (2017) 133(2):223–44. 10.1007/s00401-016-1631-4 27766432 PMC5250666

[75] McLaughlin KA Chitnis T Newcombe J Franz B Kennedy J McArdel S Age-dependent B cell autoimmunity to a myelin surface antigen in pediatric multiple sclerosis. J Immunol (2009) 183(6):4067–76. 10.4049/jimmunol.0801888 19687098 PMC2795331

[76] Krishnamoorthy G Saxena A Mars LT Domingues HS Mentele R Ben-Nun A Myelin-specific T cells also recognize neuronal autoantigen in a transgenic mouse model of multiple sclerosis. Nat Med (2009) 15(6):626–32. 10.1038/nm.1975 19483694

[77] Fissolo N Haag S de Graaf KL Drews O Stevanovic S Rammensee HG Naturally presented peptides on major histocompatibility complex I and II molecules eluted from central nervous system of multiple sclerosis patients. Mol Cell Proteomics (2009) 8(9):2090–101. 10.1074/mcp.M900001-MCP200 19531498 PMC2742442

[78] Muraro PA Kalbus M Afshar G McFarland HF Martin R. T cell response to 2’,3’-cyclic nucleotide 3’-phosphodiesterase (CNPase) in multiple sclerosis patients. J Neuroimmunol (2002) 130(1–2):233–42. 10.1016/S0165-5728(02)00229-1 12225906

[79] Quintana FJ Farez MF Viglietta V Iglesias AH Merbl Y Izquierdo G Antigen microarrays identify unique serum autoantibody signatures in clinical and pathologic subtypes of multiple sclerosis. Proc Natl Acad Sci U S A (2008) 105(48):18889–94. 10.1073/pnas.0806310105 19028871 PMC2596207

[80] Terryberry JW Thor G Peter JB. Autoantibodies in neurodegenerative diseases: antigen-specific frequencies and intrathecal analysis. Neurobiol Aging (1998) 19(3):205–16. 10.1016/S0197-4580(98)00049-9 9661995

[81] Andersson M Yu M Soderstrom M Weerth S Baig S Solders G Multiple MAG peptides are recognized by circulating T and B lymphocytes in polyneuropathy and multiple sclerosis. Eur J Neurol (2002) 9(3):243–51. 10.1046/j.1468-1331.2002.00391.x 11985632

[82] Bielekova B Sung MH Kadom N Simon R McFarland H Martin R. Expansion and functional relevance of high-avidity myelin-specific CD4+ T cells in multiple sclerosis. J Immunol (2004) 172(6):3893–904. 10.4049/jimmunol.172.6.3893 15004197

[83] Olsson T Sun J Hillert J Hojeberg B Ekre HP Andersson G Increased numbers of T cells recognizing multiple myelin basic protein epitopes in multiple sclerosis. Eur J Immunol (1992) 22(4):1083–7. 10.1002/eji.1830220431 1372558

[84] de Rosbo NK Kaye JF Eisenstein M Mendel I Hoeftberger R Lassmann H The myelin-associated oligodendrocytic basic protein region MOBP15-36 encompasses the immunodominant major encephalitogenic epitope(s) for SJL/J mice and predicted epitope(s) for multiple sclerosis-associated HLA-DRB1*1501. J Immunol (2004) 173(2):1426–35. 10.4049/jimmunol.173.2.1426 15240739

[85] Wallstrom E Khademi M Andersson M Weissert R Linington C Olsson T. Increased reactivity to myelin oligodendrocyte glycoprotein peptides and epitope mapping in HLA DR2(15)+ multiple sclerosis. Eur J Immunol (1998) 28(10):3329–35. 10.1002/(SICI)1521-4141(199810)28:10<3329::AID-IMMU3329>3.3.CO;2-2 9808202

[86] Weissert R Kuhle J de Graaf KL Wienhold W Herrmann MM Muller C High immunogenicity of intracellular myelin oligodendrocyte glycoprotein epitopes. J Immunol (2002) 169(1):548–56. 10.4049/jimmunol.169.1.548 12077287

[87] Probstel AK Dornmair K Bittner R Sperl P Jenne D Magalhaes S Antibodies to MOG are transient in childhood acute disseminated encephalomyelitis. Neurology (2011) 77(6):580–8. 10.1212/WNL.0b013e318228c0b1 21795651

[88] Johns TG Bernard CC. Binding of complement component Clq to myelin oligodendrocyte glycoprotein: a novel mechanism for regulating CNS inflammation. Mol Immunol (1997) 34(1):33–8. 10.1016/S0161-5890(97)00005-9 9182874

[89] Bartos A Fialova L Soukupova J Kukal J Malbohan I Pit’ha J. Elevated intrathecal antibodies against the medium neurofilament subunit in multiple sclerosis. J Neurol (2007) 254(1):20–5. 10.1007/s00415-006-0185-0 17508137

[90] Trotter JL Hickey WF van der Veen RC Sulze L. Peripheral blood mononuclear cells from multiple sclerosis patients recognize myelin proteolipid protein and selected peptides. J Neuroimmunol (1991) 33(1):55–62. 10.1016/0165-5728(91)90034-5 1711538

[91] Schmidt S Linington C Zipp F Sotgiu S de Waal Malefyt R Wekerle H Multiple sclerosis: comparison of the human T-cell response to S100 beta and myelin basic protein reveals parallels to rat experimental autoimmune panencephalitis. Brain (1997) 120(Pt 8):1437–45. 10.1093/brain/120.8.1437 9278633

[92] Banki K Colombo E Sia F Halladay D Mattson DH Tatum AH Oligodendrocyte-specific expression and autoantigenicity of transaldolase in multiple sclerosis. J Exp Med (1994) 180(5):1649–63. 10.1084/jem.180.5.1649 7964452 PMC2191732

[93] Colombo E Banki K Tatum AH Daucher J Ferrante P Murray RS Comparative analysis of antibody and cell-mediated autoimmunity to transaldolase and myelin basic protein in patients with multiple sclerosis. J Clin Invest (1997) 99(6):1238–50. 10.1172/JCI119281 9077532 PMC507938

[94] Varrin-Doyer M Spencer CM Schulze-Topphoff U Nelson PA Stroud RM Cree BA Aquaporin 4-specific T cells in neuromyelitis optica exhibit a Th17 bias and recognize Clostridium ABC transporter. Ann Neurol (2012) 72(1):53–64. 10.1002/ana.23651 22807325 PMC3405197

[95] Lennon VA Wingerchuk DM Kryzer TJ Pittock SJ Lucchinetti CF Fujihara K A serum autoantibody marker of neuromyelitis optica: distinction from multiple sclerosis. Lancet (2004) 364(9451):2106–12. 10.1016/S0140-6736(04)17551-X 15589308

[96] Bruck W Popescu B Lucchinetti CF Markovic-Plese S Gold R Thal DR Neuromyelitis optica lesions may inform multiple sclerosis heterogeneity debate. Ann Neurol (2012) 72(3):385–94. 10.1002/ana.23621 23034911

[97] Sato DK Callegaro D Lana-Peixoto MA Waters PJ de Haidar Jorge FM Takahashi T Distinction between MOG antibody-positive and AQP4 antibody-positive NMO spectrum disorders. Neurology (2014) 82(6):474–81. 10.1212/WNL.0000000000000101 24415568 PMC3937859

[98] Do LD Chanson E Desestret V Joubert B Ducray F Brugiere S Characteristics in limbic encephalitis with anti-adenylate kinase 5 autoantibodies. Neurology (2017) 88(6):514–24. 10.1212/WNL.0000000000003586 28062719

[99] Lai M Hughes EG Peng X Zhou L Gleichman AJ Shu H AMPA receptor antibodies in limbic encephalitis alter synaptic receptor location. Ann Neurol (2009) 65(4):424–34. 10.1002/ana.21589 19338055 PMC2677127

[100] Antoine JC Absi L Honnorat J Boulesteix JM de Brouker T Vial C Antiamphiphysin antibodies are associated with various paraneoplastic neurological syndromes and tumors. Arch Neurol (1999) 56(2):172–7. 10.1001/archneur.56.2.172 10025422

[101] Lancaster E Huijbers MG Bar V Boronat A Wong A Martinez-Hernandez E Investigations of caspr2, an autoantigen of encephalitis and neuromyotonia. Ann Neurol (2011) 69(2):303–11. 10.1002/ana.22297 21387375 PMC3059252

[102] Monstad SE Nostbakken JK Vedeler CA. CRMP5 antibodies found in a patient with limbic encephalitis and myasthenia gravis. J Neurol Neurosurg Psychiatry (2009) 80(2):241–2. 10.1136/jnnp.2008.149336 19151024

[103] Trotter JL Hendin BA Osterland CK Cerebellar degeneration with Hodgkin disease. An immunological study. Arch Neurol (1976) 33(9):660–1. 10.1001/archneur.1976.00500090066014 962649

[104] Dale RC Merheb V Pillai S Wang D Cantrill L Murphy TK Antibodies to surface dopamine-2 receptor in autoimmune movement and psychiatric disorders. Brain (2012) 135(Pt 11):3453–68. 10.1093/brain/aws256 23065479

[105] Boronat A Gelfand JM Gresa-Arribas N Jeong HY Walsh M Roberts K Encephalitis and antibodies to dipeptidyl-peptidase-like protein-6, a subunit of Kv4.2 potassium channels. Ann Neurol (2013) 73(1):120–8. 10.1002/ana.23756 23225603 PMC3563722

[106] Petit-Pedrol M Armangue T Peng X Bataller L Cellucci T Davis R Encephalitis with refractory seizures, status epilepticus, and antibodies to the GABAA receptor: a case series, characterisation of the antigen, and analysis of the effects of antibodies. Lancet Neurol (2014) 13(3):276–86. 10.1016/S1474-4422(13)70299-0 24462240 PMC4838043

[107] Lancaster E Lai M Peng X Hughes E Constantinescu R Raizer J Antibodies to the GABA(B) receptor in limbic encephalitis with seizures: case series and characterisation of the antigen. Lancet Neurol (2010) 9(1):67–76. 10.1016/S1474-4422(09)70324-2 19962348 PMC2824142

[108] Bien CG Vincent A Barnett MH Becker AJ Blumcke I Graus F Immunopathology of autoantibody-associated encephalitides: clues for pathogenesis. Brain (2012) 135(Pt 5):1622–38. 10.1093/brain/aws082 22539258

[109] Kim J Namchuk M Bugawan T Fu Q Jaffe M Shi Y Higher autoantibody levels and recognition of a linear NH2-terminal epitope in the autoantigen GAD65, distinguish stiff-man syndrome from insulin-dependent diabetes mellitus. J Exp Med (1994) 180(2):595–606. 10.1084/jem.180.2.595 7519242 PMC2191592

[110] Carvajal-Gonzalez A Leite MI Waters P Woodhall M Coutinho E Balint B Glycine receptor antibodies in PERM and related syndromes: characteristics, clinical features and outcomes. Brain (2014) 137(Pt 8):2178–92. 10.1093/brain/awu142 24951641 PMC4107739

[111] Tanaka M Maruyama Y Sugie M Motizuki H Kamakura K Tanaka K. Cytotoxic T cell activity against peptides of Hu protein in anti-Hu syndrome. J Neurol Sci (2002) 201(1–2):9–12. 10.1016/S0022-510X(02)00157-0 12163187

[112] Dalmau J Graus F Rosenblum MK Posner JB Anti-Hu-associated paraneoplastic encephalomyelitis/sensory neuronopathy. A clinical study of 71 patients. Medicine (1992) 71(2):59–72. 10.1097/00005792-199203000-00001 1312211

[113] Sabater L Gaig C Gelpi E Bataller L Lewerenz J Torres-Vega E A novel non-rapid-eye movement and rapid-eye-movement parasomnia with sleep breathing disorder associated with antibodies to IgLON5: a case series, characterisation of the antigen, and post-mortem study. Lancet Neurol (2014) 13(6):575–86. 10.1016/S1474-4422(14)70051-1 24703753 PMC4104022

[114] Vincent A Buckley C Schott JM Baker I Dewar BK Detert N Potassium channel antibody-associated encephalopathy: a potentially immunotherapy-responsive form of limbic encephalitis. Brain (2004) 127(Pt 3):701–12. 10.1093/brain/awh077 14960497

[115] Lai M Huijbers MG Lancaster E Graus F Bataller L Balice-Gordon R Investigation of LGI1 as the antigen in limbic encephalitis previously attributed to potassium channels: a case series. Lancet Neurol (2010) 9(8):776–85. 10.1016/S1474-4422(10)70137-X 20580615 PMC3086669

[116] Dalmau J Gultekin SH Voltz R Hoard R DesChamps T Balmaceda C Ma1, a novel neuron- and testis-specific protein, is recognized by the serum of patients with paraneoplastic neurological disorders. Brain (1999) 122(Pt 1):27–39. 10.1093/brain/122.1.27 10050892

[117] Dalmau J Graus F Villarejo A Posner JB Blumenthal D Thiessen B Clinical analysis of anti-Ma2-associated encephalitis. Brain (2004) 127(Pt 8):1831–44. 10.1093/brain/awh203 15215214

[118] Sillevis Smitt P Kinoshita A De Leeuw B Moll W Coesmans M Jaarsma D Paraneoplastic cerebellar ataxia due to autoantibodies against a glutamate receptor. N Engl J Med (2000) 342(1):21–7. 10.1056/NEJM200001063420104 10620645

[119] Lancaster E Martinez-Hernandez E Titulaer MJ Boulos M Weaver S Antoine JC Antibodies to metabotropic glutamate receptor 5 in the Ophelia syndrome. Neurology (2011) 77(18):1698–701. 10.1212/WNL.0b013e3182364a44 22013185 PMC3208954

[120] Gresa-Arribas N Planaguma J Petit-Pedrol M Kawachi I Katada S Glaser CA Human neurexin-3alpha antibodies associate with encephalitis and alter synapse development. Neurology (2016) 86(24):2235–42. 10.1212/WNL.0000000000002775 27170573 PMC4909558

[121] Dalmau J Tuzun E Wu HY Masjuan J Rossi JE Voloschin A Paraneoplastic anti-N-methyl-d-aspartate receptor encephalitis associated with ovarian teratoma. Ann Neurol (2007) 61(1):25–36. 10.1002/ana.21050 17262855 PMC2430743

[122] Hiasa Y Kunishige M Mitsui T Kondo S Kuriwaka R Shigekiyo S Complicated paraneoplastic neurological syndromes: a report of two patients with small cell or non-small cell lung cancer. Clin Neurol Neurosurg (2003) 106(1):47–9. 10.1016/S0303-8467(03)00059-3 14643918

[123] Luque FA Furneaux HM Ferziger R Rosenblum MK Wray SH Schold SC Jr Anti-Ri: an antibody associated with paraneoplastic opsoclonus and breast cancer. Ann Neurol (1991) 29(3):241–51. 10.1002/ana.410290303 2042940

[124] Greenlee JE Brashear HR. Antibodies to cerebellar Purkinje cells in patients with paraneoplastic cerebellar degeneration and ovarian carcinoma. Ann Neurol (1983) 14(6):609–13. 10.1002/ana.410140603 6360029

[125] Jaeckle KA Graus F Houghton A Cardon-Cardo C Nielsen SL Posner JB. Autoimmune response of patients with paraneoplastic cerebellar degeneration to a Purkinje cell cytoplasmic protein antigen. Ann Neurol (1985) 18(5):592–600. 10.1002/ana.410180513 2416270

[126] Bataller L Wade DF Graus F Stacey HD Rosenfeld MR Dalmau J. Antibodies to Zic4 in paraneoplastic neurologic disorders and small-cell lung cancer. Neurology (2004) 62(5):778–82. 10.1212/01.WNL.0000113749.77217.01 15007130 PMC2574539

[127] Lennon VA Kryzer TJ Pittock SJ Verkman AS Hinson SR. IgG marker of optic-spinal multiple sclerosis binds to the aquaporin-4 water channel. J Exp Med (2005) 202(4):473–7. 10.1084/jem.20050304 16087714 PMC2212860

[128] Kitley J Woodhall M Waters P Leite MI Devenney E Craig J Myelin-oligodendrocyte glycoprotein antibodies in adults with a neuromyelitis optica phenotype. Neurology (2012) 79(12):1273–7. 10.1212/WNL.0b013e31826aac4e 22914827

[129] Asavapanumas N Ratelade J Verkman AS Unique neuromyelitis optica pathology produced in naive rats by intracerebral administration of NMO-IgG. Acta Neuropathol (2014) 127(4):539–51. 10.1007/s00401-013-1204-8 24190619 PMC3954950

[130] Phuan PW Ratelade J Rossi A Tradtrantip L Verkman AS. Complement-dependent cytotoxicity in neuromyelitis optica requires aquaporin-4 protein assembly in orthogonal arrays. J Biol Chem (2012) 287(17):13829–39. 10.1074/jbc.M112.344325 22393049 PMC3340190

[131] McKeon A Pittock SJ Paraneoplastic encephalomyelopathies: pathology and mechanisms. Acta Neuropathol (2011) 122(4):381–400. 10.1007/s00401-011-0876-1 21938556

[132] Graus F Titulaer MJ Balu R Benseler S Bien CG Cellucci T A clinical approach to diagnosis of autoimmune encephalitis. Lancet Neurol (2016) 15(4):391–404. 10.1016/S1474-4422(15)00401-9 26906964 PMC5066574

[133] Planaguma J Leypoldt F Mannara F Gutierrez-Cuesta J Martin-Garcia E Aguilar E Human N-methyl d-aspartate receptor antibodies alter memory and behaviour in mice. Brain (2015) 138(Pt 1):94–109. 10.1093/brain/awu310 25392198 PMC4285189

[134] Moscato EH Peng X Jain A Parsons TD Dalmau J Balice-Gordon RJ. Acute mechanisms underlying antibody effects in anti-N-methyl-d-aspartate receptor encephalitis. Ann Neurol (2014) 76(1):108–19. 10.1002/ana.24195 24916964 PMC4296347

[135] Castillo-Gomez E Kastner A Steiner J Schneider A Hettling B Poggi G The brain as immunoprecipitator of serum autoantibodies against N-methyl-d-aspartate receptor subunit NR1. Ann Neurol (2016) 79(1):144–51. 10.1002/ana.24545 26505629

[136] Kim TJ Lee ST Moon J Sunwoo JS Byun JI Lim JA Anti-LGI1 encephalitis is associated with unique HLA subtypes. Ann Neurol (2017) 81(2):183–92. 10.1002/ana.24860 28026029

[137] Gastaldi M Thouin A Vincent A. Antibody-mediated autoimmune encephalopathies and immunotherapies. Neurotherapeutics (2016) 13(1):147–62. 10.1007/s13311-015-0410-6 26692392 PMC4720680

[138] De Santis G Caniatti L De Vito A De Gennaro R Granieri E Tola MR A possible paraneoplastic neuromyelitis optica associated with lung cancer. Neurol Sci (2009) 30(5):397–400. 10.1007/s10072-009-0112-0 19565183

[139] Al-Harbi T Al-Sarawi A Binfalah M Dermime S. Paraneoplastic neuromyelitis optica spectrum disorder associated with stomach carcinoid tumor. Hematol Oncol Stem Cell Ther (2014) 7(3):116–9. 10.1016/j.hemonc.2014.06.001 24954081

[140] Reagan TJ Freiman IS. Multiple cerebral gliomas in multiple sclerosis. J Neurol Neurosurg Psychiatry (1973) 36(4):523–8. 10.1136/jnnp.36.4.523 4354397 PMC494404

[141] Currie S Urich H. Concurrence of multiple sclerosis and glioma. J Neurol Neurosurg Psychiatry (1974) 37(5):598–605. 10.1136/jnnp.37.5.598 4365433 PMC494706

[142] Paydarfar D de la Monte SM Case records of the Massachusetts General Hospital. Weekly clinicopathological exercises. Case 12-1997. A 50-year-old woman with multiple sclerosis and an enlarging frontal-lobe mass. N Engl J Med (1997) 336(16):1163–71. 10.1056/NEJM199704173361608 9099662

[143] Blachere NE Orange DE Santomasso BD Doerner J Foo PK Herre M T cells targeting a neuronal paraneoplastic antigen mediate tumor rejection and trigger CNS autoimmunity with humoral activation. Eur J Immunol (2014) 44(11):3240–51. 10.1002/eji.201444624 25103845 PMC4296561

[144] Hauser SL Waubant E Arnold DL Vollmer T Antel J Fox RJ B-cell depletion with rituximab in relapsing-remitting multiple sclerosis. N Engl J Med (2008) 358(7):676–88. 10.1056/NEJMoa0706383 18272891

[145] Jarius S Aboul-Enein F Waters P Kuenz B Hauser A Berger T Antibody to aquaporin-4 in the long-term course of neuromyelitis optica. Brain (2008) 131(Pt 11):3072–80. 10.1093/brain/awn240 18945724 PMC2577801

[146] Ishiura H Matsuda S Higashihara M Hasegawa M Hida A Hanajima R Response of anti-NMDA receptor encephalitis without tumor to immunotherapy including rituximab. Neurology (2008) 71(23):1921–3. 10.1212/01.wnl.0000336648.43562.59 19047564

[147] Hauser SL Bar-Or A Comi G Giovannoni G Hartung HP Hemmer B Ocrelizumab versus interferon beta-1a in relapsing multiple sclerosis. N Engl J Med (2017) 376(3):221–34. 10.1056/NEJMoa1601277 28002679

[148] Montalban X Hauser SL Kappos L Arnold DL Bar-Or A Comi G Ocrelizumab versus placebo in primary progressive multiple sclerosis. N Engl J Med (2017) 376(3):209–20. 10.1056/NEJMoa1606468 28002688

[149] Molnarfi N Schulze-Topphoff U Weber MS Patarroyo JC Prod’homme T Varrin-Doyer M MHC class II-dependent B cell APC function is required for induction of CNS autoimmunity independent of myelin-specific antibodies. J Exp Med (2013) 210(13):2921–37. 10.1084/jem.20130699 24323356 PMC3865476

[150] Forsthuber TG Shive CL Wienhold W de Graaf K Spack EG Sublett R T cell epitopes of human myelin oligodendrocyte glycoprotein identified in HLA-DR4 (DRB1*0401) transgenic mice are encephalitogenic and are presented by human B cells. J Immunol (2001) 167(12):7119–25. 10.4049/jimmunol.167.12.7119 11739534

[151] Investigators CT Coles AJ Compston DA Selmaj KW Lake SL Moran S Alemtuzumab vs. interferon beta-1a in early multiple sclerosis. N Engl J Med (2008) 359(17):1786–801. 10.1056/NEJMoa0802670 18946064

[152] Cohen JA Coles AJ Arnold DL Confavreux C Fox EJ Hartung HP Alemtuzumab versus interferon beta 1a as first-line treatment for patients with relapsing-remitting multiple sclerosis: a randomised controlled phase 3 trial. Lancet (2012) 380(9856):1819–28. 10.1016/S0140-6736(12)61769-3 23122652

[153] Coles AJ Twyman CL Arnold DL Cohen JA Confavreux C Fox EJ Alemtuzumab for patients with relapsing multiple sclerosis after disease-modifying therapy: a randomised controlled phase 3 trial. Lancet (2012) 380(9856):1829–39. 10.1016/S0140-6736(12)61768-1 23122650

[154] Azzopardi L Cox AL McCarthy CL Jones JL Coles AJ. Alemtuzumab use in neuromyelitis optica spectrum disorders: a brief case series. J Neurol (2016) 263(1):25–9. 10.1007/s00415-015-7925-y 26477020

[155] Pittock SJ Lennon VA McKeon A Mandrekar J Weinshenker BG Lucchinetti CF Eculizumab in AQP4-IgG-positive relapsing neuromyelitis optica spectrum disorders: an open-label pilot study. Lancet Neurol (2013) 12(6):554–62. 10.1016/S1474-4422(13)70076-0 23623397

